# Neural Processing of Facial Attractiveness and Romantic Love: An Overview and Suggestions for Future Empirical Studies

**DOI:** 10.3389/fpsyg.2022.896514

**Published:** 2022-06-14

**Authors:** Ryuhei Ueda

**Affiliations:** ^1^Institute for the Future of Human Society, Kyoto University, Kyoto, Japan; ^2^Center for Information and Neural Networks, National Institute of Information and Communications Technology, Osaka, Japan

**Keywords:** romantic love, facial attractiveness, social decision-making, social cognition, neuroimaging

## Abstract

Romantic love is universally observed in human communities, and the manner in which a person chooses a long-term romantic partner has been a central question in studies on close relationships. Numerous empirical psychological studies have demonstrated that facial attractiveness greatly impacts initial romantic attraction. This close link was further investigated by neuroimaging studies showing that both viewing attractive faces and having romantic thoughts recruit the reward system. However, it remains unclear how our brains integrate perceived facial attractiveness into initial romantic attraction. In addition, it remains unclear how our brains shape a persistent attraction to a particular person through interactions; this persistent attraction is hypothesized to contribute to a long-term relationship. After reviewing related studies, I introduce methodologies that could help address these questions.

## Introduction

Our lives are filled with social relationships. Some close relationships, including romantic relationships, might last for years or even decades. In the initiation of a long-term relationship, deliberate thinking is likely required to infer the personal traits of a person that one encounters. Contrary to this intuition, experimental studies have demonstrated that personality inferences from facial appearance are performed very rapidly and strongly influence the perceiver’s attitude toward the person (Todorov et al., 2008, 2013). This review focuses on the cognitive process in the context of the initiation of a romantic relationship; more specifically, how is the perceived facial attractiveness of a potential partner integrated into the initial romantic attraction? In this review, I employ the definition of the term “attraction” provided by Montoya and Horton (2014): “a person’s immediate and positive affective and/or behavioral response to a specific individual, a response that is influenced by the person’s cognitive assessments.”

### Close Link Between Facial Attractiveness and Romantic Attraction

Romantic love is observed in nearly all societies (Jankowiak and Fischer, 1992) and is thought to be deeply connected to human mate selection (e.g., Fisher, 2004; Walum and Young, 2018). For these reasons, extensive studies on close relationships have been devoted to understanding how people evaluate potential partners. Early experimental studies reported that facial attractiveness could predict initial romantic attraction on actual dates (e.g., Walster et al., 1966; Byrne et al., 1970). This close link has been focused on by evolutionary psychologists working on human mate selection (e.g., Buss, 1989; Buss and Schmitt, 1993). Buss (1989) conducted a cross-cultural survey and observed significantly higher desirability for partners’ physical attractiveness by males than by females in almost all countries. Based on the findings, the authors hypothesized that several features shown in attractive faces (e.g., smooth skin, good muscle tone, lustrous hair, and full lips) could signal higher fertility and reproductive value or good health. A meta-analysis conducted by Feingold (1990) observed greater value placed on physical attractiveness of the female partners by males, although the sex difference was larger when examining self-reports than examining behavior. Based on these findings, Eastwick et al. (2014) predicted that sex differences in predictability of facial attractiveness of the partner on romantic evaluation could vary depending on the relationship stages. To test this prediction, the authors conducted a meta-analysis of studies that investigated romantic evaluation on an opposite-sex individual whom the participant has at least met face to face. The authors indicated that facial attractiveness of the partner strongly predicts romantic evaluation both for males and females to a similar degree, suggesting that the sex differences in ideal partner preference might emerge only in the stage of forming impressions of an ideal partner before a face-to-face interaction. This study and another study (Zsok et al., 2017) further suggested that this link might be stronger in the initiation stage than during the postformation stage of a long-term relationship.

We might think that we consider personal traits other than facial attractiveness when choosing an “ideal” partner. In line with this intuition, online dating services, which have rapidly expanded and surpassed meetings through friends in the United States this decade (Rosenfeld et al., 2019), usually require access to user profiles to facilitate searches for ideal partners. However, this intuitive view has been challenged by empirical evidence showing that stated mate preferences do not successfully predict initial romantic attraction in a live face-to-face interaction (e.g., Todd et al., 2007; Joel et al., 2017). Furthermore, a large dataset obtained from a commercial dating service indicated that while physically observable attributes, including attractiveness, strongly influenced romantic evaluation, harder-to-observe attributes had only small effects (Kurzban and Weeden, 2005). This almost exclusive influence of facial attractiveness on romantic evaluation likely reflects that people tend to pursue short-term partners. However, this view has been contradicted by a study showing that both males and females generally pursue not short-term but long-term partners in live face-to-face interactions, even at a younger age (Asendorpf et al., 2011). Based on these empirical findings, Eastwick et al. (2014) pointed out that the heavy reliance on descriptive profiles that are part of online dating services might reflect a misunderstanding of the information that people use to evaluate potential partners. In sum, facial attractiveness rapidly and strongly induces initial romantic attraction even when people pursue long-term partners, and people may tend to underestimate the impact of facial attractiveness.

### Neurobiological Basis of Perceiving Facial Attractiveness

**Figure 1:**
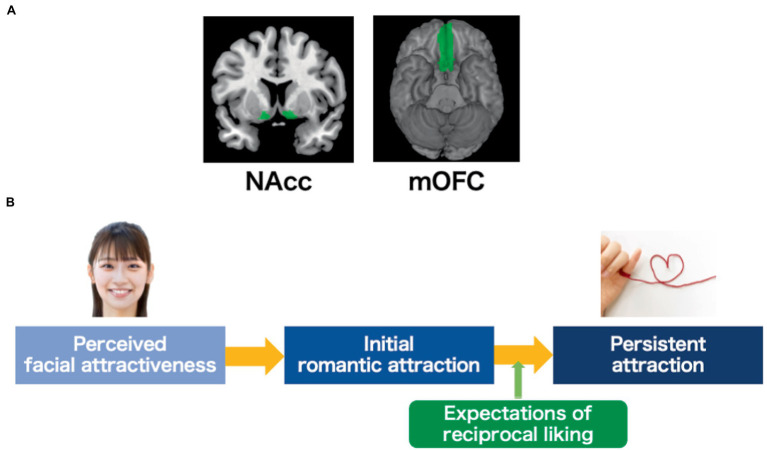
**(A)** Main brain regions involved in the euphoria of viewing attractive faces: the nucleus accumbens (NAcc) and medial orbitofrontal cortex (mOFC). The images were created using the WFU_PickAtlas (Maldjian et al., 2003, 2004) and MRIcron (Rorden et al., 2007). **(B)** Schematic model of integration between perceived facial attractiveness and initial romantic attraction. A persistent attraction to a particular person must be shaped through interactions even before a long-term relationship is formed, which could be modulated by expectations of reciprocal liking. Copyright: ImageNavi, republished with permission.

How does the human brain process attractive faces, which may inspire romantic interest? Since the 2000s, neuroimaging studies have aimed to identify the neurobiological basis for the perception of facial attractiveness. Early functional magnetic resonance imaging (fMRI) studies revealed that viewing attractive faces induces neural activity in reward-related brain regions, mainly the nucleus accumbens (NAcc) and medial orbitofrontal cortex (mOFC) (e.g., Aharon et al., 2001; O’Doherty et al., 2003; Figure 1A). Engagement of these regions is hypothesized to reflect the subjective reward value of faces. This prediction has been supported by subsequent studies showing that the sexual orientation of the perceiver modulates neural responses in the mOFC when viewing attractive male and female faces (Kranz and Ishai, 2006; Ishai, 2007). Other studies have provided evidence to support the model indicating that rapid and automatic engagement of the NAcc to presented faces conveys a preference signal to the mOFC underlying deliberative evaluation (Kim et al., 2007; Chatterjee et al., 2009).

### Romantic Evaluation of Potential Partners Modulated by Relationship Status

Neuroimaging studies have revealed that engagement of the reward system, in which attractive faces are more valued, elicits romantic interest. After the formation of a long-term relationship, however, attractive alternative partners who may threaten the relationship may be devalued (Johnson and Rusbult, 1989; Simpson et al., 1990). Meyer et al. (2011) examined neural correlates of this derogation effect. The authors observed increased activation in the control-related prefrontal regions during successful derogation of attractive alternative partners in the absence of cognitive load, which was negatively correlated with activation in the ventral striatum. These results suggest that when cognitive resources were available, recruiting the prefrontal region could contribute to the deliberate regulation of attraction to alternative partners. In contrast, engagement of prefrontal regions during successful derogation was not observed under cognitive load, suggesting that derogation could implicitly occur without requiring cognitive resources. While early studies on relationship maintenance mainly focused on explicit and deliberate aspects of regulation (Ritter et al., 2010; Pronk et al., 2011), an increased focus has been placed on encompassing such implicit and automatic aspects into the theoretical framework (Linardatos and Lydon, 2011; Finkel and Eastwick, 2015; Karremans et al., 2015; Lydon and Karremans, 2015; Pronk and Righetti, 2015; Ueda et al., 2017, 2018).

Subjective preference for attractive faces may also be modulated by the relationship status of the potential partner; that is, people tend to devalue attractive opposite-sex individuals who are in a relationship with a significant other. This kind of derogation seems to reflect the avoidance of “mate poaching” (Schmitt and Buss, 2001; Schmitt et al., 2004). Our previous work reported that individuals characterized by greater mOFC responses demonstrated a greater preference for hypothetical partners in a relationship with a significant other (Ueda et al., 2017), suggesting that this region might underpin the deliberate evaluation of potential partners (Kim et al., 2007; Chatterjee et al., 2009).

### Behavioral and Neural Alterations After Building a Close Relationship

A long-term close relationship can be characterized by a persistent attraction to the partner both in human and nonhuman animals (Fisher et al., 2016; Walum and Young, 2018). For example, romantically involved individuals tend to exhibit a more positive evaluation of their partner’s attractiveness (Murray et al., 1996; Murray and Holmes, 1997) and give less attention to alternative partners (Miller, 1997; Maner et al., 2008, 2009). These behavioral tendencies have been hypothesized to contribute to relationship maintenance (Linardatos and Lydon, 2011; Finkel and Eastwick, 2015; Karremans et al., 2015; Lydon and Karremans, 2015; Pronk and Righetti, 2015).

Those observations then raise the following question: how does the reward system shape a persistent attraction to a particular person? A neurobiological study on monogamous prairie voles provided evidence that revealed the relevant neural mechanisms (Aragona et al., 2006). The authors observed that the two dopamine (DA) receptor subtypes in the rostral shell of the NAcc have opposing roles in regulating pair bonding in male monogamous prairie voles. Specifically, the activation of DA D2-type receptors facilitated partner preference, whereas the activation of D1-type receptors not only did not induce partner preference but also prevented partner preference. The authors also observed increased D1-type receptors within the NAcc in pair-bonded males compared with nonpair-bonded males after 2 weeks of exposure to a female but not after only 24 h. This upregulation effect was not observed within the caudate putamen, and the other subtype, D2-type receptors, did not differ in either brain region. Given that 24 h of mating resulted in a partner preference, the authors argued that reorganization of the NAcc might not be necessary for the initial formation of the partner preference and that increased D1-type receptors in the NAcc might reflect a more fully established pair-bond, which could contribute to pair-bond maintenance.

Neural mechanisms underpinning pair-bond development in prairie voles are now considered to be precisely understood, and those findings are expected to be helpful for understanding the human mating system (Fisher et al., 2016; Walum and Young, 2018). This view has been partly supported by human neuroimaging studies, which have consistently reported engagement of the reward system, including the NAcc, when people have romantic thoughts about their committed partner (Bartels and Zeki, 2000, 2004; Aron et al., 2005; Xu et al., 2011; Acevedo et al., 2012; Takahashi et al., 2015). Our recent work using a decoding approach (see below) further demonstrated that the spatial activation patterns of the NAcc related to a romantic partner could be discriminated from those related to other opposite-sex individuals (Ueda and Abe, 2021). This finding is consistent with the hypothesis that the formation of a distinct neural representation of a long-term partner underpins a partner preference, which could contribute to relationship maintenance (Walum and Young, 2018).

While human neuroimaging studies have thus far been successful in identifying neural correlates underpinning the euphoria of viewing attractive faces and having romantic thoughts about a committed partner, it remains unclear how our brains integrate perceived facial attractiveness into initial romantic attraction. In addition, while prairie vole studies suggest that neural correlates underpinning a persistent attraction to a particular person must be shaped even before a long-term relationship is formed, we are still far from understanding the comparable neural mechanisms in humans (Figure 1B). In the following sections, I provide suggestions for future empirical studies to bridge this gap.

## Discussion: Acquisition of Empirical Evidence Demonstrating the Neural Processing of Initial Romantic Attraction

### Combining Neuroimaging With an Ecologically Valid Task

To provide empirical evidence to address these issues, an ecologically valid experimental paradigm is needed first and foremost. Specifically, the assessment of romantic evaluation is more suitable in a real-life situation than in a hypothetical situation. The “speed-dating” paradigm fulfills this requirement to address questions relevant to initial attraction and relationship development in a live face-to-face interaction (Finkel et al., 2007; Eastwick and Finkel, 2008b). In the standard version of this paradigm, male and female participants who want to build a long-term relationship have a few minutes of interaction with each speed-dating partner, and at the end of each date, they are asked to evaluate their romantic interest (e.g., “Are you interested in meeting again this person?”). Male and female participants who showed mutual romantic interest in a speed-dating event are given an opportunity to meet again after completion of the study, although researchers are required to deal with several ethical issues before launching the study (e.g., potential risk of experiencing social rejection or social awkwardness, or very serious interpersonal troubles; Finkel et al., 2007).

Finkel et al. (2007) pointed out that while most studies had used ratings of hypothetical targets to assess romantic attraction (for reviews, Cooper and Sheldon, 2002; Montoya et al., 2018), some studies investigated this matter in a natural setting (e.g., Walster et al., 1966; Byrne et al., 1970; Bernstein et al., 1983; see also Feingold, 1990). Finkel et al. (2007) argued that in comparison with those real-life settings, the speed-dating paradigm has benefits other than ecological validity for evaluating initial romantic attraction. Specifically, unlike a traditional longer date with one partner, each participant simultaneously evaluates many partners and is evaluated by them in a few minutes during speed-dating events, which allows researchers to examine the degree to which features of a given interaction are derived from one partner, the other partner, or the unique dynamics between the two partners. As we see later, this feature also allows researchers to test the learning process during romantic evaluation (Cooper et al., 2014). Furthermore, Finkel et al. (2007) argued that a large array of experimental manipulations, such as comparing the effects of different date durations or disclosing personal information before starting a date on romantic evaluation, can be incorporated into the paradigm. Studies using this paradigm have mainly examined the predictability of factors related to ideal partner preference (e.g., facial attractiveness, body attractiveness, education, and income), which were originally identified through evaluation of hypothetical targets (e.g., Buss, 1989), on romantic evaluation in face-to-face interactions (e.g., Kurzban and Weeden, 2005; Fisman et al., 2006; Eastwick et al., 2007; Todd et al., 2007; Eastwick and Finkel, 2008a; Asendorpf et al., 2011; Joel et al., 2017).

**Figure 2:**
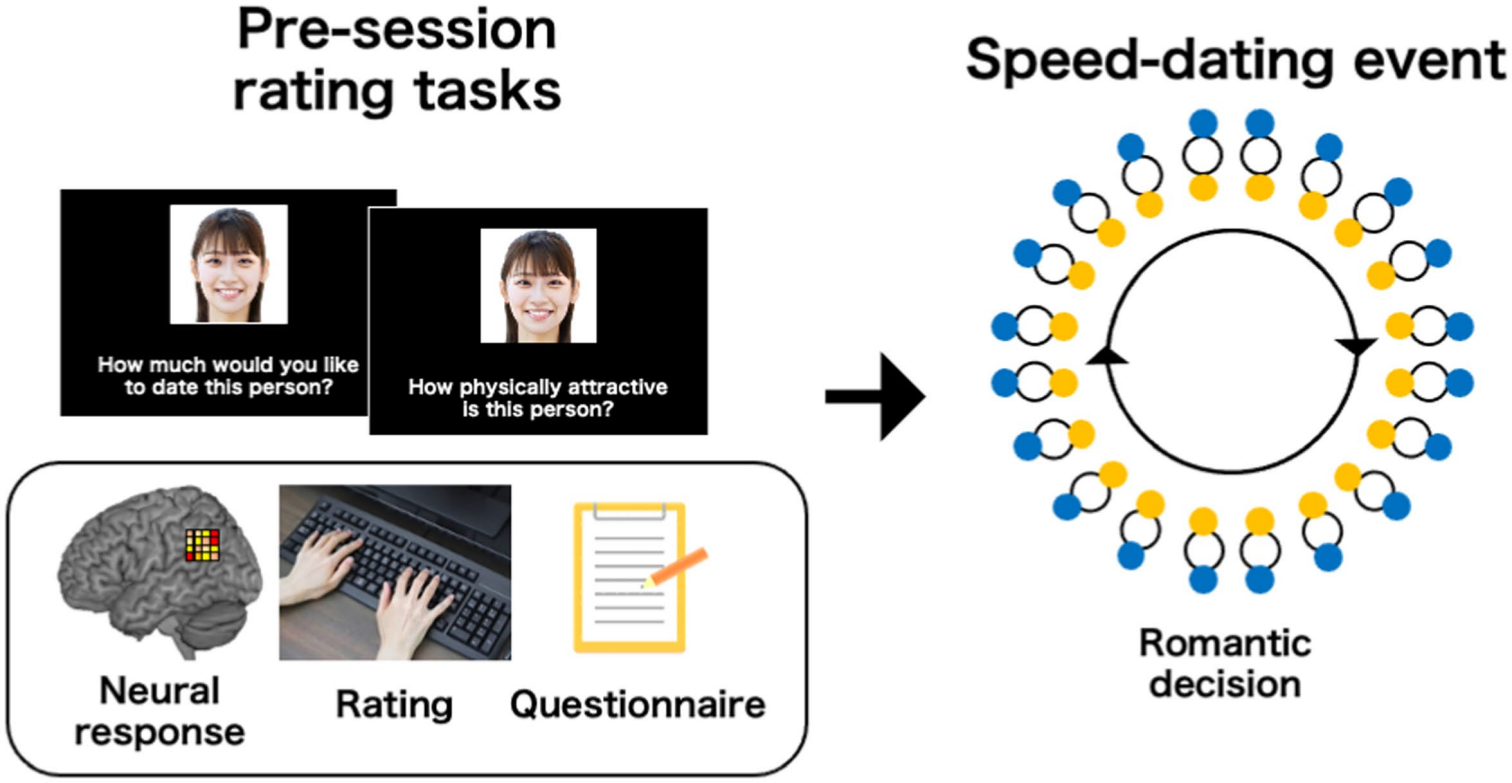
Schematic illustration of the experimental procedure used in Cooper et al. (2012), which combined fMRI with a speed-dating paradigm. Prior to speed-dating events, researchers can collect neural data while initially viewing each speed-dating partner. Other rating data, including perceived facial attractiveness or questionnaire data, can also be recorded. In subsequent speed-dating events, participants met each other and had short conversations. At the end of each date, participants were asked to make romantic decisions about whether they wanted to see each partner again. Combining neural and behavior data that are recorded in a session with romantic decisions allowed us to assess the predictability of neural activation or self-reported ideal partner traits on the romantic decisions. Copyright: ImageNavi, republished with permission.

Cooper et al. (2012) combined fMRI with this paradigm to test whether the neural activity that occurs while initially viewing potential partners’ faces could predict romantic decisions in subsequent speed dating events (Figure 2). The authors found that neural activity in the paracingulate cortex was predictive of both subsequent romantic decisions and perceived facial attractiveness, suggesting that this region might be involved in the formation of an initial rapid romantic evaluation. The authors also observed that perceived facial attractiveness ratings were represented in the reward system, including in the striatum and ventromedial prefrontal cortex; however, more importantly, neural activation in the reward system was not predictive of subsequent decisions, implying distinctive neural processes during romantic evaluation.

Recent neuroimaging studies using the speed-dating paradigm have further provided intriguing findings, including synchronization of brain activities between the two people who are interacting (Yuan and Liu, 2022), the automatic formation of a “reflected impression” on the speed-dating partner (i.e., the degree to which participants expect the partner to positively view them; Ito et al., 2020), and similarities of functional connectivity profiles obtained from resting-state fMRI data that are predictive of the compatibility of the impressions between the two partners (Kajimura et al., 2021). On the other hand, no previous studies have directly revealed how our brains integrate perceived facial attractiveness into initial romantic attraction. I introduce several analytical methodologies in the following sections that could be useful in addressing this issue.

### Disentanglement of the Overlapping Function

Cooper et al. (2012) observed overlapping neural activity associated with facial attractiveness and initial romantic attraction in the paracingulate cortex. Their findings lead to the question of how this region processes each piece of information; that is, does this region process the initial romantic attraction of the potential partner in a different way than that which occurs when only viewing attractive faces? This question is difficult to address through conventional fMRI analysis, which aims to identify brain regions showing a greater response to the experimental conditions than to a control condition. A decoding approach using multivoxel pattern analysis (MVPA) could allow us to test this question by disentangling overlapping neural activity within the region. This method uses a machine-learning technique to decode mental states or cognitive processes from the spatial patterns of neural activity in a target region, thus allowing better interpretation of overlapping functional activations (Norman et al., 2006; Peelen and Downing, 2007). The simplest case of the decoding approach assumes that if one cognitive process induces specific neural activity patterns, the trained classifier can accurately discriminate activation patterns between the conditions. If the paracingulate cortex encodes the formation of initial romantic attraction for a particular person in a different way from perceived facial attractiveness, the neural activity patterns should be distinguishable from those related to individuals with attractive faces who are not selected as potential romantic partners in subsequent decisions. This investigation might help elucidate the early neural processing during romantic evaluation, i.e., how our brains shape preference for a partner at first sight.

One may think that engagement of the paracingulate cortex might also be observed in an evaluation process other than romantic evaluation, including choosing a friend based on the person’s facial appearance or evaluation of the likeability on nonsocial objects. In other words, investigation of the specificity of neural processing would be needed. For this kind of investigation with higher-order representational space across multiple dimensions, another type of multivariate method, representational similarity analysis (RSA; Kriegeskorte et al., 2008; Kriegeskorte and Kievit, 2013), may be a suitable approach. RSA quantifies the (dis)similarity of the spatial patterns of neural activity among stimuli within the target region based on the Pearson correlation coefficient or other measures. The (dis)similarity among stimuli can be summarized as a representation (dis)similarity matrix, and then researchers can assess the relatedness between the neural (dis)similarity and the stimulus features. This standard procedure of RSA is well summarized in the review by Popal et al. (2019). In the case of extending findings by Cooper et al. (2012), by including additional conditions (e.g., viewing same-sex unfamiliar individuals or scenery pictures) and/or combining with data from control rating tasks (e.g., evaluation of perceived roundness of faces; Kim et al., 2007), researchers may test the functional specificity of neural processing of preference for a partner at first sight (i.e., examining whether significant association of neural activity patterns with ratings across stimuli in the paracingulate cortex is observed only for romantic evaluation). MVPA including RSA that focuses on neural activity patterns across multiple voxels has an advantage over individual-voxel-based conventional method in increased sensitivity to the cognitive states (Norman et al., 2006). In addition, as described above, disentanglement of overlapping neural activity within the region could contribute to revealing the representational content (Popal et al., 2019). Another strong point of RSA is that it allows a direct comparison of data obtained from different species in a common representation space (Kriegeskorte et al., 2008). Given the importance of bridging the gap with the findings obtained from monogamous prairie vole studies, this investigation would also be valuable.

### Modulation of Initial Romantic Attraction Through Interaction

This review has thus far focused on initial romantic attraction. In the last section, I provide suggestions for investigating an advanced question: how could initial romantic attraction be modulated through the subsequent interaction? As described above, a close relationship can be characterized by a persistent attraction to a particular person, and studies have suggested specific neural mechanisms in the reward system (Aragona et al., 2006; Fisher et al., 2016; Walum and Young, 2018; Ueda and Abe, 2021).

Although little is known about the pertinent dynamic neural process, the computational modeling approach used in Cooper et al. (2014) may contribute to revealing this issue. In Cooper et al. (2014), following the completion of speed-dating events, some participants were evaluated by fMRI while they “learned” about their own desirability by receiving information indicating romantic interest from their speed-dating partners. To investigate neural responses associated with learned expectations about partner decisions, the authors implemented a reinforcement learning model. This model assumed that participants learned during the scan how likely partners were to express romantic interest in them and that the expectations would be updated based on the degree to which the expectations were violated at each trial (i.e., prediction error). This kind of computational approach has been widely used to describe the cognitive process of various social behaviors (Dunne and O’Doherty, 2013). Subtraction analysis of neuroimaging data demonstrated significantly greater responses to unexpected romantic interest expressed by a speed-dating partner in the posterior superior temporal sulcus. Furthermore, neural responses to a partner’s decision were positively correlated with unsigned prediction errors based on the learning model in the medial prefrontal cortex. These two brain areas have been associated with the mentalizing process: encoding and updating beliefs about the intentions and feelings of others (Frith, 2007). While the model used in Cooper et al. (2014) focused on learning of the person’s own desirability, this approach might also be applicable to describe the updating of the subjective preference to each partner through repeated interaction instead of through one-shot speed dating events.

Modulation of initial romantic attraction may involve other cognitive functions, such as emotion processing. People tend to like those who seem to like them (i.e., expectations of reciprocal liking; e.g., Aron et al., 1989; Riela et al., 2010), and fear of rejection could decrease their willingness to approach a potential partner (e.g., Bernstein et al., 1983). Cooper et al. (2014) reported greater neural responses in the anterior cingulate to rejection by a partner in whom a participant was romantically interested. This region is engaged when one experiences “social pain” caused by romantic rejection (Kross et al., 2011). Another study has shown that learning of an attractive potential partner’s interest has a greater impact on romantic evaluation (Greitemeyer, 2010), implying that there is interplay between perceived facial attractiveness and emotion processing during interactions. Top-down cognitive control supported by the dorsolateral prefrontal cortex (Heatherton and Wagner, 2011) may also be involved in the modulation process. Specifically, successful devaluation of a potential partner who demonstrates less interest might elicit increased neural responses in the dorsolateral prefrontal cortex and decreased responses in the reward system. Connectivity analyses assessed by psycho-physiological interaction (PPI; Friston et al., 1997) or dynamic causal modeling (DCM; Friston et al., 2003) would allow us to directly test this hypothesis.

## Conclusion

How we choose a long-term romantic partner has been a central question in studies on close relationships. In the past two decades, human neuroimaging studies have identified engagement of the reward system in the euphoria of viewing attractive faces, which may induce initial romantic attraction. Studies combining neuroimaging with speed dating have further identified specific neural responses that predict subsequent romantic decisions. These findings lead to further questions: How do our brains integrate signals related to perceived facial attractiveness into initial romantic attraction? How do our brains shape a persistent attraction to a particular person through interactions? Future studies will address these issues by elucidating the neural representations, dynamic alterations, and computational process underpinning the behavior.

## Author Contributions

The author confirms being the sole contributor of this work and approves it for publication.

## Funding

This work was supported by JSPS KAKENHI grant number JP20K20157.

## Conflict of Interest

The author declares that the research was conducted in the absence of any commercial or financial relationships that could be construed as a potential conflict of interest.

## Publisher’s Note

All claims expressed in this article are solely those of the authors and do not necessarily represent those of their affiliated organizations, or those of the publisher, the editors and the reviewers. Any product that may be evaluated in this article, or claim that may be made by its manufacturer, is not guaranteed or endorsed by the publisher.
